# Collective Behaviour without Collective Order in Wild Swarms of Midges

**DOI:** 10.1371/journal.pcbi.1003697

**Published:** 2014-07-24

**Authors:** Alessandro Attanasi, Andrea Cavagna, Lorenzo Del Castello, Irene Giardina, Stefania Melillo, Leonardo Parisi, Oliver Pohl, Bruno Rossaro, Edward Shen, Edmondo Silvestri, Massimiliano Viale

**Affiliations:** 1 Istituto Sistemi Complessi, Consiglio Nazionale delle Ricerche, UOS Sapienza, Rome, Italy; 2 Dipartimento di Fisica, Università Sapienza, Rome, Italy; 3 Initiative for the Theoretical Sciences, The Graduate Center, City University of New York, New York, New York, United States of America; 4 Dipartimento di Informatica, Università Sapienza, Rome, Italy; 5 Dipartimento di Fisica, Università di Roma 3, Rome, Italy; 6 Dipartimento di Scienze per gli Alimenti la Nutrizione e l'Ambiente, Università degli Studi di Milano, Milano, Italy; Eotvos Lorand University, Hungary

## Abstract

Collective behaviour is a widespread phenomenon in biology, cutting through a huge span of scales, from cell colonies up to bird flocks and fish schools. The most prominent trait of collective behaviour is the emergence of global order: individuals synchronize their states, giving the stunning impression that the group behaves as one. In many biological systems, though, it is unclear whether global order is present. A paradigmatic case is that of insect swarms, whose erratic movements seem to suggest that group formation is a mere epiphenomenon of the independent interaction of each individual with an external landmark. In these cases, whether or not the group behaves truly collectively is debated. Here, we experimentally study swarms of midges in the field and measure how much the change of direction of one midge affects that of other individuals. We discover that, despite the lack of collective order, swarms display very strong correlations, totally incompatible with models of non-interacting particles. We find that correlation increases sharply with the swarm's density, indicating that the interaction between midges is based on a metric perception mechanism. By means of numerical simulations we demonstrate that such growing correlation is typical of a system close to an ordering transition. Our findings suggest that correlation, rather than order, is the true hallmark of collective behaviour in biological systems.

## Author Summary

Our perception of collective behaviour in biological systems is closely associated to the emergence of order on a group scale. For example, birds within a flock align their directions of motion, giving the stunning impression that the group is just one organism. Large swarms of midges, mosquitoes and flies, however, look quite chaotic and do not exhibit any group ordering. It is therefore unclear whether these systems are true instances of collective behaviour. Here we perform the three dimensional tracking of large swarms of midges in the field and find that swarms display strong collective behaviour despite the absence of collective order. In fact, we discover that the capability of swarms to collectively respond to perturbations is surprisingly large, comparable to that of highly ordered groups of vertebrates.

## Introduction

Intuition tells us that a system displays collective behaviour when all individuals spontaneously do the same thing, whatever this thing may be. We surely detect collective behaviour when all birds in a flock fly in the same direction and turn at the same time [1], as well as when all spins in a magnet align, giving rise to a macroscopic magnetization [2], [3]. On the other hand, we would not say that there is any collective behaviour going on in a gas, despite the large number of molecules. The concept of collective behaviour seems therefore closely linked to that of emergent collective order, or synchronization. Indeed, explaining how order spontaneously arises from local inter-individual interactions has been one of the major issues in the field [4]–[6].

The case of insect swarms is tricky in this respect. Several species in the vast taxonomic order Diptera (flies, mosquitoes, midges) form big swarms consisting largely of males, whose purpose is to attract females [7], [8]. Swarming therefore has a key reproductive function and, in some cases, relevant health implications, the obvious, but not unique, example being that of the malaria mosquito, *Anopheles gambiae* [9]–[11]. It is well-known that swarms form in proximity of some visual marker, like a water puddle, or a street lamp [7]. Swarming insects seem to fly independently around the marker, without paying much attention to each other (see Video S1). For this reason, the question of whether swarms behave as truly collective systems is debated [4], [12]. In fact, it has even been suggested that in Diptera there is no interaction between individuals within the swarm and therefore no collective behaviour at all [13], [14]. Although other studies observed local coordination between nearest neighbours [15], [16], it remains controversial whether and to what extent collective patterns emerge over the scale of the whole group. Clarifying this issue is a central goal in swarms containment [17], [18]. In absence of quantitative evidence telling the contrary, the hypothesis that external factors, as the marker, are the sole cause of swarming and that no genuine collective behaviour is present, is by far the simplest explanation.

We must, however, be careful in identifying collective behaviour with collective order. There are systems displaying important collective effects both in their ordered *and* in their disordered phase. An example is that of a ferromagnet near the critical temperature 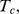 i.e. the temperature below which a spontaneous magnetization emerges: the collective response of the system to an external perturbation is as strong in the disordered phase slightly above 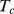 as it is in the ordered phase slightly below 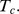 In fact, once below the critical temperature, increasing the amount of order *lowers* the collective response [2], [3]. Similarly, in animal behaviour it is possible to conceive cases in which individuals coordinate their behavioural reactions to environmental stimuli, rather than the behaviours themselves; conversely we may expect that a group too heavily ordered, i.e. with a very large behavioural polarization, may respond poorly to perturbations, because of an excessive behavioural inertia. Hence, although certainly one of its most visible manifestations, emergent order is not necessarily the best probe of collective behaviour.

The crucial task for living groups is not simply to achieve an ordered state, but to respond collectively to the environmental stimuli. For this to happen, correlation must be large, namely individuals must be able to influence each other's behavioural changes on a group scale. The question then arises of whether correlation in biological systems is a consequence of collective order or whether it can be sustained even in absence of order. The question is relevant because the way individuals in a group synchronize their behavioural fluctuations (correlation) is possibly a more general mechanism than the synchronization of behaviour itself (order). All experimental studies performed up to now, however, concerned highly synchronized groups (as bird flocks, fish shoals and marching locusts [19]–[21]), which displayed both order and correlation. Hence, the question of whether or not order and correlations are two sides of the same phenomenon remained open until now. Here, we attempt to give an answer to this question by experimentally studying large swarms of insects in the field. As we will show, despite the lack of collective order, we do find strong correlations, indicating that in biological systems collective behaviour and group-level coordination do not require order to be sustained.

## Results

### Experiments and tracking

We perform an experimental study of swarms of wild midges in the field. Midges are small non-biting flies belonging to the order Diptera, suborder Nematocera (Diptera:Chironomidae and Diptera:Ceratopogonidae - see Methods). The body length of the species we study is in the range 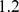
–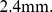 Swarms are found at sunset, in the urban parks of Rome, typically near stagnant water. As noted before [7], we find that swarms form above natural or artificial landmarks. Moving the landmark leads to an overall displacement of the swarm. The swarms we studied range in size between 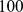 and 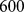 individuals (see Table S1 in Text S1).

**Figure 1:**
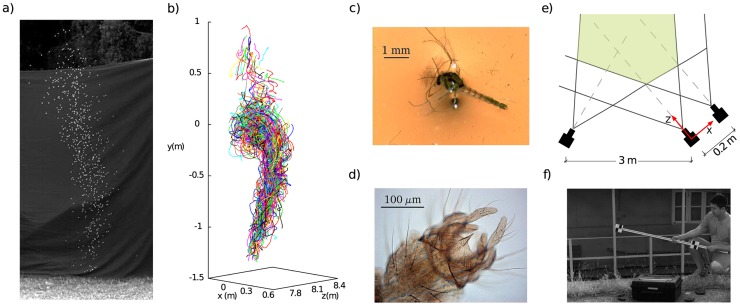
Experiment. **a**: A natural swarm of midges (*Cladotanytarsus atridorsum*, Diptera:Chironomidae), in Villa Ada, Rome. The digital image of each midges is, on average, a 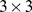 pixels light object against a dark background. **b**: The 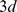 trajectories reconstructed for the same swarm as in **a**. Individual trajectories are visualized for a short time (roughly 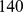 frames 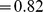
sec), to avoid visual overcrowding (see also Video S1 and S2). **c**: A microscope image of an adult male of *Cladotanytarsus atridorsum*. **d**: A detailed view of the hypopygium, used for species identification (see Methods); the same midge as in **c**. **e**: Scheme of the experimental set-up. Three synchronized cameras recording at 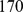 frames per second are used. Two cameras 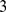 m apart are used as the stereoscopic pair for the three dimensional reconstruction. The third one is used to reduce tracking ambiguities and resolve optical occlusions. Three dimensional trajectories are reconstructed in the reference frame of the right stereoscopic camera. **f**: The mutual geometric positions and orientations of the cameras are retrieved by taking several pictures of a known target. The accuracy we achieve in the determination of the mutual camera orientation is of the order of 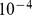 radians.

To reconstruct the 3d trajectories of individual insects we use three synchronized cameras shooting at 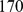 frames-per-seconds (trifocal technique – Fig. 1e and Methods). Our apparatus does not perturb the swarms in any way. The technique is similar to the one we used for starling flocks [22], with one notable difference. To reach the desired experimental accuracy we need to know the mutual geometric relations between the three cameras very accurately. In the case of flocks, this could be achieved only by an *a priori* alignment of the cameras. In the case of swarms, though, we proceed differently. After each swarm acquisition, we pin down the geometry of the camera system by taking multiple images of a calibrated target (Fig. 1f). This procedure is so accurate that the error in the 3d reconstruction is dominated by the image segmentation error due to the pixel resolution. If we assume this to be equal to 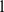 pixel (typically it is smaller than that because midges occupy many pixels), we make an error of 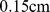 in the determination of the distance between two points 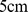 apart from each other (a reference value for nearest neighbour distance). The absolute error is the same for more distant points, making the relative precision of our apparatus even higher. This accuracy makes the determination of the correlation functions we study here very reliable.

The 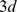-tracking of each midge is performed by using the recursive global optimization method described in [23]. This recursive algorithm dramatically reduces the complexity of the tracking problem, effectively overcoming the limit of other tracking methods [24], [25], and allowing the reconstruction of large swarms, up to 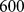 midges, for long time, up to 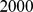 frames. Sample 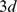 reconstructions are shown in Fig. 1b and in Video S2. Compared to previous field [11], [15], [26] and lab [27]–[29] studies, data collected and analysed in the present work have the advantage to span among swarms of different sizes and densities.

### Lack of collective order

**Figure 2:**
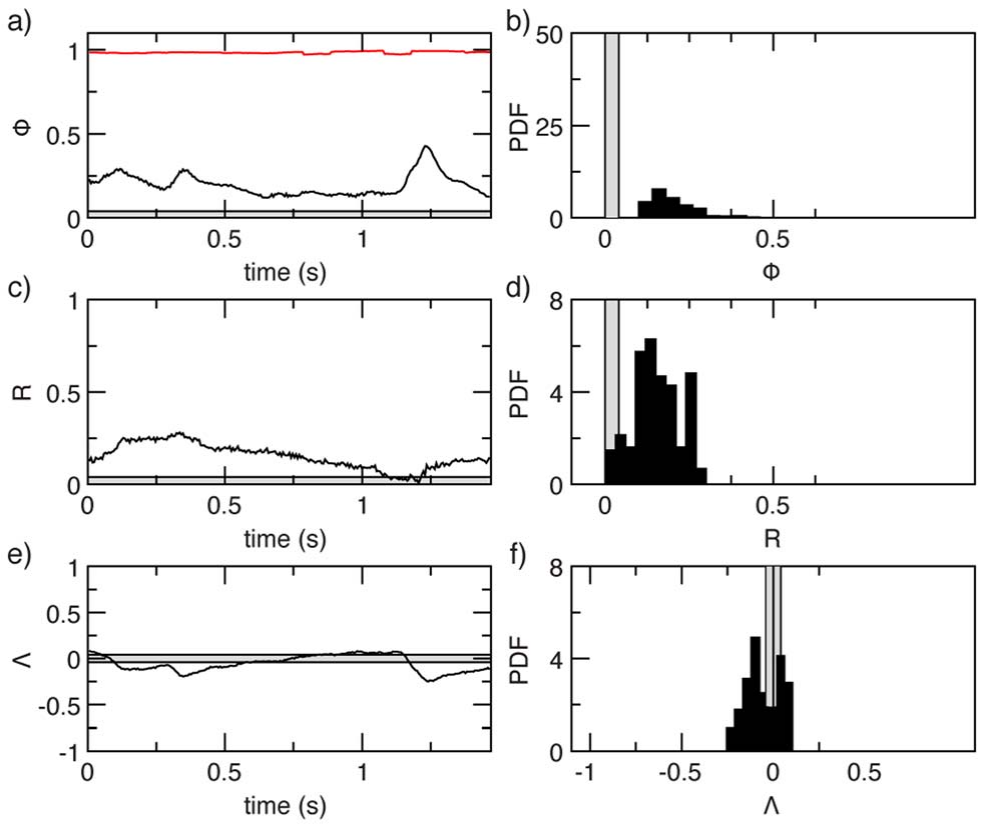
Natural swarms lack global order. Order parameters in a typical natural swarm. In all panels the grey band around zero is the expected amplitude of the fluctuations in a completely uncorrelated system. In the left panels we report the time series of the order parameters, in the right panels their probability distributions. **Top**: The alignment order parameter, known as polarization, 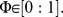 In red we report the reference value of the polarization in a flock of starlings. **Middle**: Rotational order parameter, 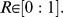 **Bottom**: Dilatational order parameter, 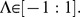

Swarms are in a disordered phase. The standard order parameter normally used in collective behaviour is the polarization, 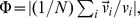 where 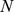 is the number of midges in the swarm and 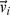 is the velocity of insect 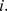 The polarization measures the degree of alignment of the directions of motion; it is a positive quantity and its maximum value is 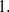 The average polarization over all swarms is quite small, 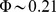 (see Fig. 2 and Table S1 in Text S1). As a reference, in starling flocks we find 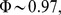 on average [19]. The probability distributions of the polarization fully confirms the swarms' lack of translational order and the stark difference with flocks (Fig. 2). Clearly, swarms are not in a polarized state. Translation is not the only possible collective mode, though. For example, it is well-known that fish schools can produce rotating (milling) configurations. Moreover, a group can expand/contract, giving rise to dilatational (or pulsive) collective modes. For this reason we have defined and measured also a rotational and a dilatational order parameter (see Methods). We find, however, that these quantities too have very small values (Fig. 2). The time series, on the other hand, show that the order parameters can have rare, but strong fluctuations, during which their value may become significantly larger than that of an uncorrelated system (Fig. 2). These large fluctuations are a first hint that non-trivial correlations are present.

### Correlation

The connected correlation function measures to what extent the change in behaviour of individual 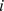 is correlated to that of individual 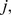 at distance 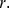 Correlation is the most accessible sign of the presence of interaction between the members of a group. The absence of interaction implies the absence of correlation. Conversely, the presence of correlation implies the presence of an effective interaction (see Text S1, Section I). Correlation can be measured for different quantities, but in the case of midges, as with birds and other moving animals, the principal quantity of interest is the direction of motion. To compute the connected correlation we first need to introduce the velocity fluctuations, namely the individual velocity subtracted of the overall motion of the group, 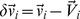 (see Methods for the detailed definition of 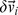 and 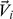). This fluctuation is a dimensional quantity, hence it is unsuitable to compare the correlation in natural vs numerical systems, as we shall do later on. We therefore introduce the dimensionless velocity fluctuation,
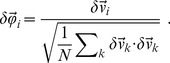
(1)


**Figure 3:**
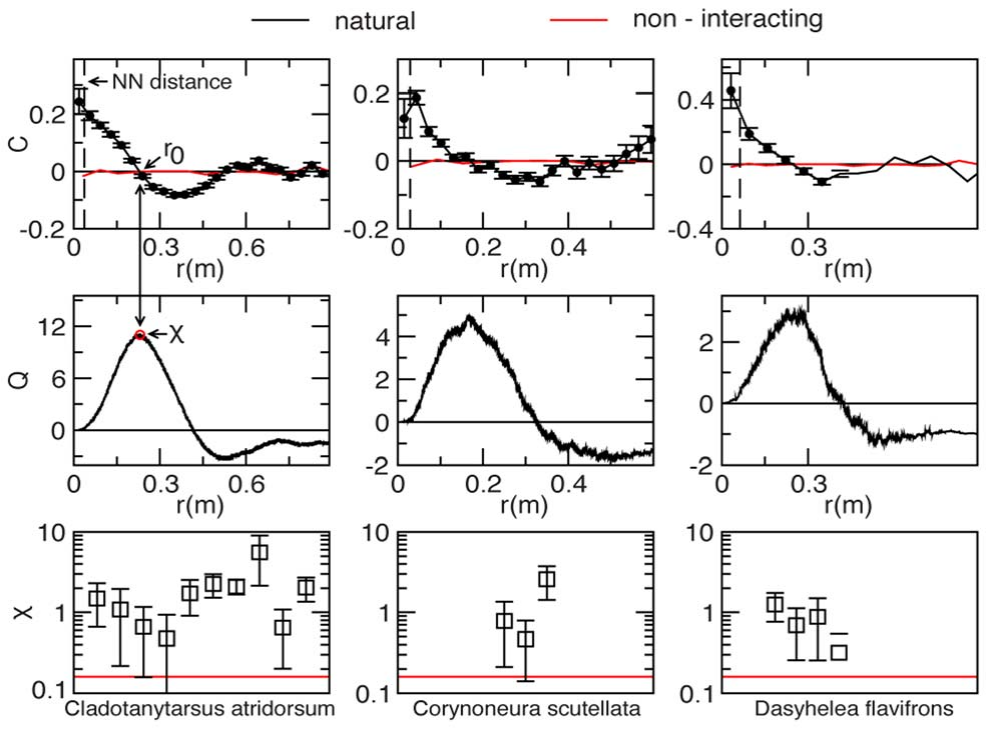
Swarms correlation. Black lines and symbols refer to natural swarms, red lines to simulations of ‘swarms’ of non-interacting particles (NHS). Each column refers to a different midge species. **Top**: Correlation function 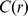 as a function of the distance at one instant of time. The dashed vertical line marks the average nearest neighbour distance, 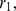 for that swarm. The correlation length, 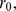 is the first zero of the correlation function. Red: correlation function in the NHS case. The value of 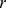 for the NHS has been rescaled to appear on the same scale as natural distances. Each natural swarm is compared to a NHS with the same number of particles. **Middle**: Cumulative correlation, 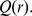 This function reaches a maximum 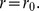 The value of the integrated correlation at its maximum, 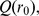 is the susceptibility 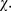 **Bottom**: Numerical values of the susceptibility 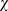 in all analysed swarms. For each swarm the value of 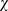 is a time average over the whole acquisition; error bars are standard deviations. Red: the average susceptibility 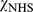 in the non-interacting case.

The connected correlation function is then given by,
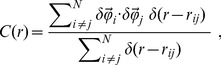
(2)where 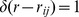 if 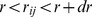 and zero otherwise, and 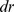 is the space binning factor. The form of 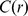 in natural swarms is reported in Fig. 3: at short distances there is strong positive correlation, indicating that midges tend to align their velocity fluctuations to that of their neighbours. After some negative correlation at intermediate distances, 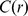 relaxes to no correlation for large distances. This qualitative form is quite typical of all species analysed (see Fig. 3). The smallest value of the distance where 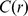 crosses zero is the correlation length, 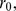 that is an estimate of the length scale over which the velocity fluctuations are correlated [19]. The average value of this correlation length over all analysed swarms is, 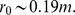 This value is about 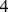 times larger than the nearest neighbours distance, whose average over all swarms is 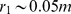 (see Fig. 3 and Table S1 in Text S1). Previous works noticed the existence of pairing manoeuvres and flight-path coordination between nearest neighbours insects [4], [15], [16]. Our results, however, indicate that midges within a natural swarm influence each other's motion far beyond their nearest neighbours.

### Susceptibility

The collective response of the swarm depends crucially on two factors: how distant in space the behavioural change of one insect affects that of another insect (spatial span of the correlation) and how strong this effect is (intensity of the correlation). To combine these two factors in one single observable we calculate the cumulative correlation up to scale 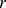,
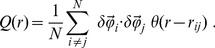
(3)where 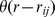 is the Heaviside function,
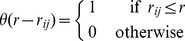



It can be shown (see Text S1, Section II) that this dimensionless function is related to the space integral of the correlation function 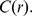 Hence, 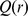 reaches a maximum where 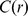 vanishes, i.e. for 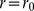 (see Fig. 3). This maximum, 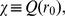 is a measure of the total amount of correlation present in the system. In statistical physics 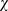 is exactly equal to the *susceptibility*, namely the response of the system to an external perturbation [30], [31]. In collective animal behaviour, we do not have a quantitative link between integrated correlation and response, so that calling 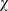 susceptibility is not strictly correct. However, if the probability distribution of the velocities is stationary, we can follow a maximum entropy approach [32] and still find that the total amount of correlation in the system, 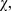 is related to the way the group responds collectively to a perturbation (see Text S1, Section II). The value of 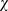 for midge swarms is reported in Fig. 3.

### Non-interacting Swarm

In order to judge how significant is the correlation function 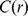 and how large is the susceptibility 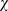 in natural swarms, we need an effective zero for these quantities, i.e. some null hypothesis baseline. As we have seen in the Introduction, the minimal assumption is that all individuals in the swarm interact with an external landmark independently from each other. Following Okubo [4] (but see also [27] and [16]), we therefore simulate a ‘swarm’ of non-interacting particles performing a random walk in a three-dimensional harmonic potential (see Methods). Visually, the group behaviour of this Non-interacting Harmonic Swarm (NHS) looks remarkably similar to that of a real swarm (see Video S2 and S3): all ‘midges’ fly around the marker and the group lacks collective order.

This similarity, however, is deceptive. In the NHS, the correlation function 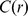 simply fluctuates around zero, with no spatial span, nor structure (Fig. 3). Moreover, the susceptibility in the NHS is extremely small, 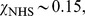 whereas the susceptibility in natural swarms is up to 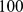 times larger than this non-interacting benchmark (Fig. 3). We conclude that swarming behaviour is *not* the mere epiphenomenon of the independent response of each insect with the marker. Despite the lack of collective order, natural swarms are strongly correlated on large length scales. There are big clusters of midges that move coherently, contributing to the ‘dancing’ visual effect of the swarm. The only way this can happen is that midges interact. What kind of interaction is that?

### Metric interaction

**Figure 4:**
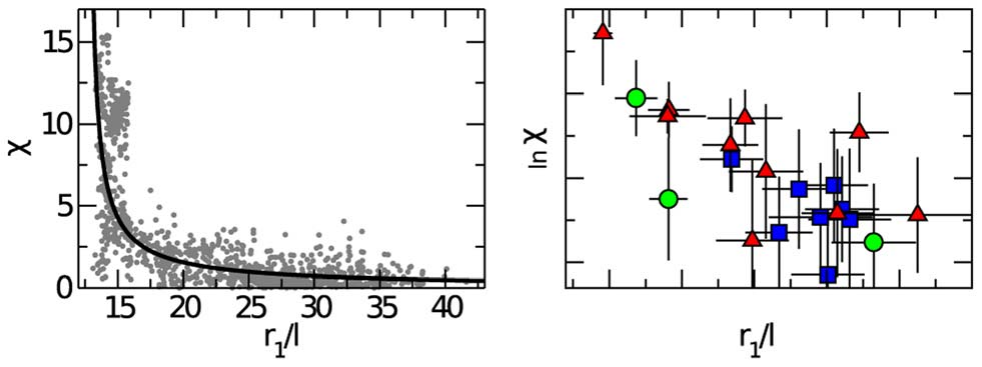
Swarms susceptibility. Left : Susceptibility 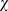 as a function of the rescaled nearest neighbour distance, 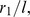 where 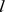 is the body length. Each point represents a single time frame of a swarming event, and all events are reported on the same plot (symbols are the same for all species). The solid line is the best fit to equation (4). **Right**: Logarithm of the average susceptibility as a function of 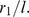 *Dasyhelea flavifrons* - blue squares; *Corynoneura scutellata* - green circles; *Cladotanytarsus atridorsum* - red triangles. The solid line represents the best fit to equation (4). Each data point represents the time average over the entire acquisition of one swarming event. Error bars indicate standard deviations.

To understand the nature of the interaction, we study the susceptibility across swarms of different densities. Interestingly, we find that 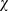 increases when the average nearest neighbour distance, 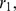 decreases (Fig. 4). Denser swarms are more correlated than sparser ones. This result indicates that midges interact through a metric perceptive apparatus: the strength of the perception decreases with the distance, so that when midges are further apart from each other (larger 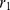) the interaction is weaker and the susceptibility 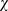 is lower. This is at variance with what happens in starling flocks: starlings interact with a fixed number of neighbours, irrespective of their nearest neighbour distance 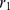 [33]; such kind of topological interaction does not depend on the group density, hence the susceptibility does not depend on the nearest neighbour distance. Fig. 4, on the other hand, shows that midges interact metrically, namely with all neighbours within a fixed metric range, 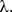 Hence, in swarms the number of interacting neighbours increases with decreasing 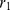 (increasing density), and as a consequence of this increased amount interaction, the system becomes also more correlated.

In a system ruled by metric interaction we expect all lengths to be measured in units of the perception range, 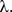 This implies that the natural variable for the susceptibility is the rescaled nearest neighbour distance, 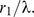 The problem is that we are considering different species of midges, likely to have different metric perception ranges. The simplest hypothesis we can make is that 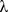 is proportional to the insect body length 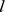 (which we can measure), so that 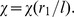 This hypothesis is confirmed by the data: the susceptibility is significantly more correlated to the variable 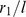 (P-value 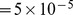) than to 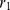 (P-value 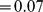 - see Methods for the definition of P-value). The fact that the natural variable is 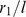 is a further indication that the interaction in swarms is based on a metric mechanism.

The difference in the nature of the interaction between flocking birds and swarming midges (topological vs. metric) is possibly due to the significant differences between vertebrates and arthropods. Topological interaction, namely tracking a fixed number of neighbours irrespective of their distance, requires a level of cognitive elaboration of the information [33] more sophisticated than a metric interaction, where the decay of the effective force is merely ruled by the physical attenuation of the signal with increasing metric distance. In other words, within a metric mechanism the range of the interaction is fixed by a perceptive cut-off, rather than a cognitive one. Metric interaction is known to be more fragile than topological one against external perturbations [33], and indeed it is far more likely to observe the dispersion of a swarm in the field than that of a flock. This may be the reason why the presence of an external marker is crucial for the swarming behaviour of midges [13].

### Correlation without order

The experimental observations of a non-trivial connected correlation and of a large susceptibility indicate that midges are effectively interacting with each other by acting on their directions of motion. This does not exclude, of course, that other types of interaction are present. First of all, the empirical observation that the swarm uses a visual marker as a reference for maintaining its mean spatial position, strongly suggests that each individual interacts with the marker. Besides, it is certainly possible that effective positional attraction-repulsion forces between midges, as those described in [34], exist. However, the directional correlations indicate that insects are *also* effectively interacting by adjusting their velocities. Moreover, the fact that these correlations are positive for short distances means that midges tend to *align* their direction of motion. This fact may seem surprising, because alignment interactions typically lead to the formation of ordered (polarized) groups, which is clearly not the case for midges. Swarms are disordered, and yet interacting and highly correlated systems. Is this a paradox?

In fact, it is not. An alignment interaction does not *per se* lead to global order in the group. In all models where imitation of the neighbours is present, the onset of long-range order depends on the value of some key tuning parameter. In a ferromagnet, this parameter is the temperature 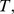 namely the amount of noise affecting the interaction between the neighbouring spins. At high temperature the system is in a disordered state, whereas by lowering 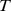 one reaches a critical temperature below which an ordering transition occurs. In models of active matter there is another parameter tuning the transition between disorder and order, that is density or, equivalently, nearest neighbour distance: the system gets ordered once the nearest neighbour distance falls below some transition value. The crucial point is that, in general, the correlation of the system tends to be very large *around* the transition point, irrespective of whether the system is in the ordered or in the disordered phase. Hence, even a disordered system can display large correlations, provided that it is not too far from an ordering transition. In what follows, we want to show that this is indeed what happens with midge swarms.

### Vicsek model

**Figure 5:**
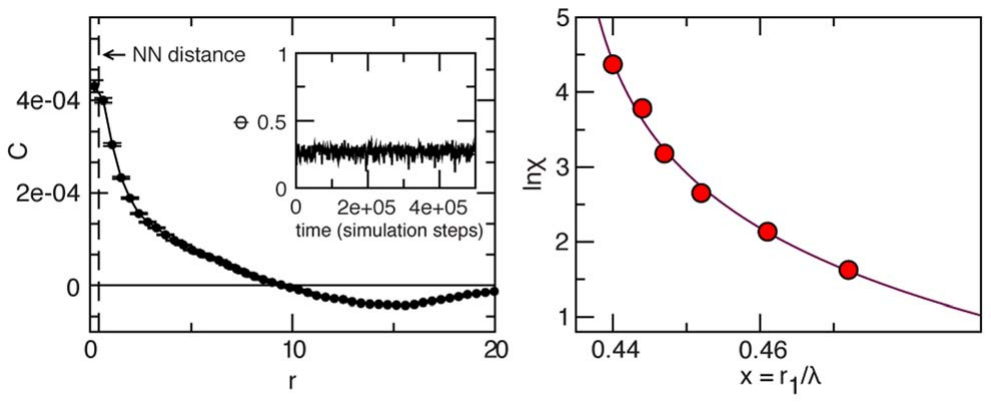
Vicsek model. Three-dimensional Vicsek model in a central potential. **Left**: Correlation function 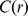 in the disordered phase, 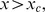 but close to the ordering transition. The dashed line is the nearest neighbour distance. Inset: polarization as a function of time. For this value of 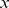 the system is disordered. **Right**: Logarithm of the susceptibility as a function of the rescaled nearest neighbour distance, 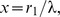 where 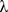 is the metric interaction range. The solid line represents the best fit to equation (4). Error bars are smaller than symbols' size.

The simplest model based on alignment interaction that predicts an order-disorder transition on changing the density is the Vicsek model of collective motion [35]. In this model each individual tends to align its direction of motion to that of the neighbours within a *metric* perception range, 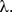 The rescaled nearest neighbour distance, 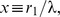 is the control parameter: for low noise, the model predicts a transition from a disordered phase (low polarization) at high values of 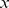 (low density), to an ordered phase (large polarization) at low values of 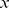 (high density) [35]–[37]. We numerically study the Vicsek model in three dimensions. As we have seen, real swarms hold their average position with respect to a marker; to reproduce this behavioural trait we introduce an harmonic attraction force that each individual experiences towards the origin (see Methods). Also in central potential the model displays an ordering transition: at large density, for 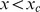 the system is ordered and it has large polarization (Video S4). On the other hand, the polarization is low in the disordered phase, 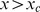 (Fig. 5). However, the correlation function is non-trivial when 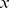 is sufficiently close to 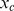 (Fig. 5), indicating the existence of large clusters of correlated individuals, which can be clearly detected in Video S5. We calculate the susceptibility 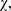 in the same manner as we did for natural swarms, in the disordered phase, 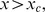 and find a clear increase of 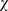 on lowering 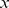 (Fig. 5).

This increase of the susceptibility is coherent with the existence of an ordering transition at 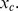 It has been shown that, unless 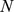 is much larger than the values analysed here, the transition in the Vicsek model is characterized by a clear second order phenomenology (the nature of the transition for 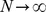 is still debated - see [37]–[39]). As a consequence, the susceptibility is expected to become very large approaching 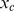 and to follow the usual scaling relation of critical phenomena [38],
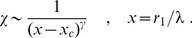
(4)


A fit to equation (4) of the 3d-Vicsek data is reported in Fig. 5, giving 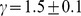 and a transition point, 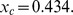 The reason for the growth of 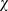 approaching 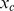 in the Vicsek model is quite intuitive. The model is metric, so that at large 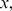 namely when the nearest neighbour distance 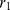 is much larger than the interaction range 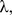 very few individuals interact with each other, and coordination is small. The smaller 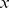 becomes, the larger the number of particles within the mutual interaction range, thus promoting the correlation of larger and larger clusters of particles. For this reason the correlation length and the susceptibility grow when the nearest neighbour distance decreases. When 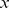 approaches its critical value, the coordinated clusters become as large as the whole system, so that the groups orders below 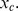


The low order parameter, the non-trivial correlation function, and especially the increase of 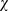 on decreasing the nearest neighbour distance, are phenomenological traits that the metric Vicsek model shares with natural swarms. We conclude that a system based solely on alignment can be in its disordered phase and yet display large correlations, as midge swarms do. It is interesting to note that by approaching the ordering transition a compound amplification of the correlation occurs: when the nearest neighbour distance, 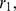 decreases, the spatial span of the correlation, 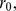 increases, so that the effective perception range in units of nearest neighbour distance, 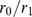 is boosted. We emphasize that we are not quantitatively fitting Vicsek model to our data. Our only aim is to demonstrate a general concept: large correlation and lack of global order can coexist even in the simplest model of nearest neighbours alignment, provided that the system is sufficiently close to an ordering transition.

### Estimating the interaction range

The consistency between our experimental data and the Vicsek model suggests that an underlying ordering transition could be present in swarms as well. An ordering transition as a function of the density has been indeed observed in laboratory experiments on locusts [21], fish [40] and in observations of oceanic fish shoals [41]. In these cases, both sides (low and high density) of the ordering transition were explored. However, midge swarms in the field are always disordered, living in the low-density/high-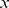 side of the transition. Locating a transition point having data on just one side of it, is a risky business. The reason why we want to do this here is because it will allow us to give a rough estimate of the metric range of interaction in midges, which can be compared with other experiments.

If a Vicsek-like ordering transition exist, we can use equation (4) to fit the swarms data for 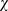 (Fig. 4). As we already mentioned, we do not know the value of the metric perception range, 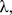 in swarms. Therefore, we use as scaling variable 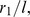 where 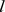 is the body length. Although the fit works reasonably well (Fig. 4), the scatter in the data is quite large; hence, given the non-linear nature of the fit, it would be unwise to pin down just one value for the parameters, and we rather report confidence intervals. The fit gives a transition point in the range, 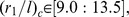 with an exponent in the range, 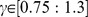 (larger exponents correspond to lower transition points).

**Figure 6:**
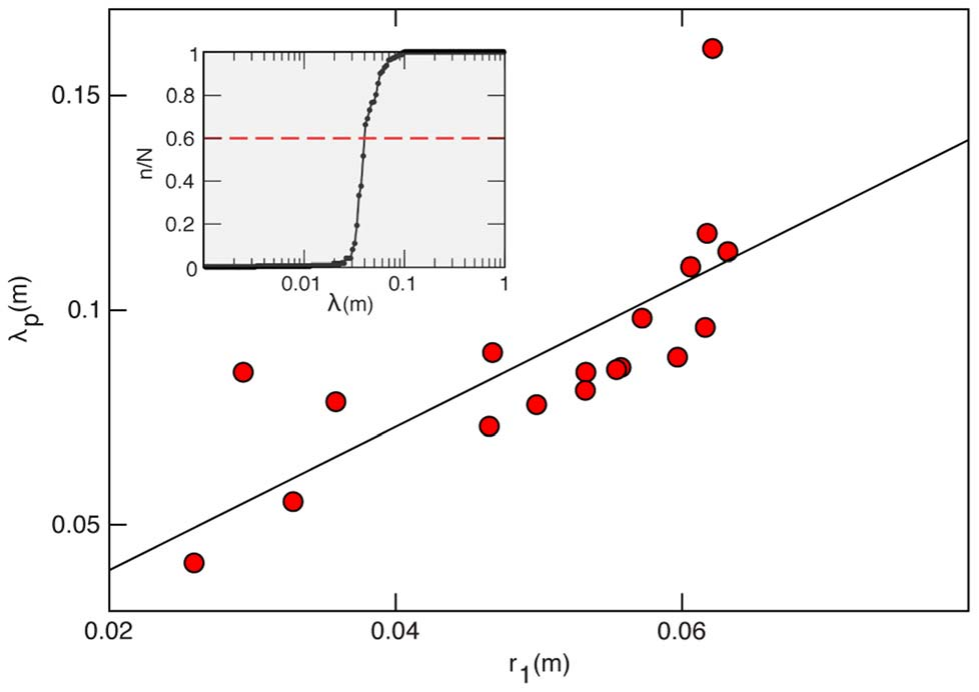
Percolation threshold. Percolation threshold 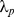 as a function of the nearest-neighbour distance in natural swarms. The linear fit (black line) gives, 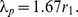 Inset: Fraction of midges belonging to the largest cluster as a function of the clustering threshold 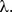 In correspondence of the percolation threshold 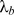 there is the formation of a giant cluster. We define 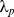 as the point where 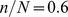 (red dashed line). Because of the sharp nature of the percolation transition, the value of 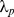 does not depend greatly on the threshold used.

Interestingly, there is an alternative way to locate the ordering transition that does not rely on the fit of 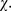 Let us establish a link between pairs of insects closer than the perception range 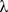 and calculate the size of the biggest connected cluster in the network. Given a swarm with nearest neighbour distance 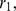 the larger 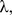 the larger this cluster. When 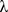 exceeds the percolation threshold, 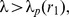 a giant cluster of the same order as the group size appears [42]. We calculate the percolation threshold in swarms (Fig. 6 and Methods) and find 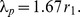 The crucial point is that varying the perception range 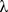 at fixed nearest neighbour distance 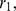 is equivalent to varying 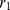 at fixed 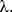 Hence, at fixed 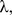 there is an equivalent percolation threshold of the nearest neighbour distance, 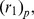 such that for 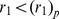 a giant cluster appears. Clearly, 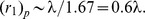 It is reasonable to hypothesise that the critical nearest neighbour distance is close to the maximal distance compatible with a connected network, given 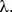 A sparser network would cause the swarm to lose bulk connectivity. Therefore, given a certain perception range 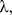 the ordering transition occurs at values of the nearest neighbour distance 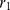 close to its percolation threshold, 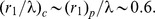


At this point we have two independent (and possibly equally unreliable) estimates of the transition point in natural swarms of midges: the first one in units of body-lengths, 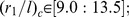 the second one in units of interaction range, 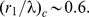 Putting the two together we finally obtain an estimate of the metric interaction range in units of body-lengths, 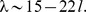 The body length of the species under consideration is in the range, 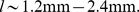 This implies a perception range of a few centimetres, 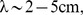 depending on the species. This crude estimate of the midge interaction range is compatible with the hypothesis that midges interact acoustically. In [43] the male-to-male auditory response in *Chironomus annularius* (Diptera:Chironomidae) was studied and it was found that the range of the response was about 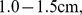 not too far from our estimate. Similar measurements in mosquitoes (Diptera:Culicidae) show that the auditory perception range is about 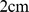 [44], which is again compatible with our determination of the interaction range in midge swarms.

## Discussion

We have shown that natural swarms of midges lack collective order and yet display strong correlations. Such correlations extends spatially much beyond the inter-individual distance, indicating the presence of significant cluster of coordinated individuals. This phenomenology is incompatible with a system of non-interacting particles whose swarming behaviour is solely due to the attraction to an external landmark. We conclude that genuine collective behaviour is present in swarms. We stress that the existence of correlation, and therefore of inter-individual interaction, is not in contradiction with the fact that a swarm almost invariably forms in proximity of a marker. The effect of the marker (external force) is merely to keep the swarm at a stationary position with respect to the environment. However, as we have shown in the case of the non-interacting swarm, this stationarity (which superficially would seems the only visible trait of swarming), cannot by itself produce the observed strong correlations. By using Vicsek model as a simple conceptual framework, we have shown that this coexistence of disorder and correlation is a general feature of systems with alignment interaction close to their ordering transition.

We should be careful in interpreting our data as proof that explicit alignment is the main interaction at work in swarms. What we can say is that non-trivial alignment correlation implies *effective* alignment interaction. However, how this effective alignment interaction is achieved in terms of sensorimotor processes is hard to tell. In fact, as we have already remarked, it is possible that models purely based on repulsion/attraction positional forces, lead to correlations similar to the ones we reported here. Hence, as always when dealing with animal behaviour, it is important to keep in mind the intrinsically *effective* nature of any interaction. The Vicsek model provides the simplest and most compelling description of collective behaviour when effective alignment is present and this fact is not hindered by the real, non-effective nature of the interaction giving rise to the observed correlations.

Our results suggest that correlation, rather than order, is the most significant experimental signature of collective behaviour. Correlation is a measure of how much and how far the behavioural change of one individual affects that of other individuals not directly interacting with it. Our data show that in swarms correlations are so strong that the effective perception range of each midge is much larger than the actual interaction range. If the change of behaviour is due to some environmental perturbations, such large correlation guarantees that the stimulus is perceived at a collective level.

A notion of collective behaviour based on correlation is more general and unifying than one based on order. For example, bird flocks and insect swarms look like completely different systems as long as we stick to collective order. However, once we switch to correlation, we understand that this big difference may be deceptive: both flocks and swarms are strongly correlated systems, in which the effective perception range, or correlation length, is far larger than the interaction range [19]. In this perspective, the striking difference in emergent order between the two systems, namely the fact that flocks move around the sky, whereas swarms do not, may be related to different ecological factors, rather than to any fundamental qualitative difference in the way these systems interact. Strong correlations similar to those found in bird flocks and midge swarms have also been experimentally measured in neural assemblies [45]. This huge diversity - birds, insects, neurons - is bewildering but fascinating, and it suggests that correlation may be a universal condition for collective behaviour, bridging the gap between vastly different biological systems.

## Methods

### Experiments

Data were collected in the field (urban parks of Rome), between May and October, in 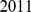 and in 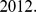 We acquired video sequences using a multi-camera system of three synchronized cameras (IDT-M5) shooting at 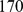 fps. Two cameras (the stereometric pair) were at a distance between 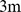 and 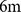 depending on the swarm and on the environmental constraints. A third camera, placed at a distance of 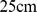 from the first camera was used to solve tracking ambiguities. We used Schneider Xenoplan 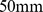 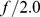 lenses. Typical exposure parameters: aperture 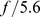, exposure time 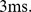 Recorded events have a time duration between 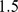 and 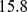 seconds. No artificial light was used. To reconstruct the 3d positions and velocities of individual midges we used the techniques developed in [23]. Wind speed was recorded. After each acquisition we captured several midges in the recorded swarm for lab analysis. A summary of all swarms data can be found in Table S1 in Text S1.

### Midge identification

We recorded swarms of midges belonging to the family Diptera:Ceratopogonidae (*Dasyhelea flavifrons*) and Diptera:Chironomidae (*Corynoneura scutellata* and *Cladotanytarsus atridorsum*). Midges belonging to the family Chironomidae were identified to species according to [46], the ones belonging to the family Ceratopogonidae were identified according to [47] and [48]. Specimens used for identification were captured with a hand net and fixed in 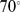 alcohol, cleared and prepared according to [49]. Permanent slides were mounted in Canada Balsam and dissected according to [50]. Species identification was based on morphology of the adult male, considering different characters, as wing venation, antennal ratio (length of apical flagellomere divided by the combined length of the more basal flagellomeres) and genitalia, which in Diptera are named hypopygium (a modified ninth abdominal segment together with the copulatory apparatus - see Fig. 1).

### Velocity fluctuations

Let 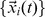 be the set of coordinates at time 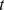 and 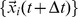 at the next time step. To simplify the notation we set 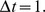 The velocity vector of insect 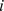 is defined as, 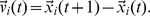 To compute the connected correlation function we need to subtract the contribution of all collective modes from the individual velocity. We identify three collective modes: translation, rotation and dilatation (expansion/contraction).

*Translation*: Let 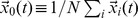 be the position of the centre of mass, and 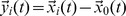 the position of the 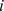-th object in the centre of mass reference frame. By subtracting the centre of mass velocity, 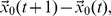 from the individual velocity, 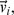 we obtain the translation-subtracted fluctuation,
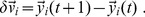
(5)


*Rotation*: The optimal rotation about the origin is defined [51] as the 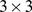 orthogonal matrix 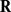 which minimizes the quantity 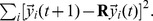 By subtracting the overall translation and rotation, the velocity fluctuation is,
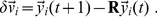
(6)


*Dilatation*: The optimal dilatation is defined [51] as the scalar 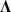 that minimizes the quantity 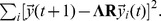 After subtracting the optimal translation, rotation and dilatation, the velocity fluctuation is finally given by,
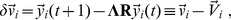
(7)where with 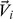 we have indicated the contribution to the velocity of 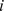 of all three collective modes.

### Rotation and dilatation order parameters

The rotational order parameter is defined as,
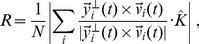
(8)where 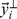 is the projection of 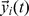 on the plane orthogonal to the axis of rotation, the operator 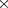 indicates the cross product, and 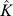 is a unit vector in the direction of the axis of rotation. In (8), 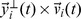 is the angular momentum of midge 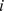 with respect to the axis 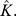 In a perfectly coherent rotation, all individuals would have angular momenta parallel to the axis, so that 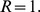 In a non-coherent system, some of the projections of the angular momentum on 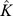 would be positive and some negative, so 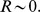 Note that 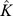 is the axis of rotation defined in the previous section, computed using Kabsch algorithm [51].

The dilatational order parameter is defined as,
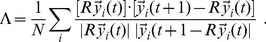
(9)



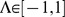
 and it measures the degree of coherent expansion (positive 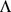) and contraction (negative 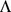) of the swarm. In a perfectly coherent expansion/contraction 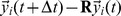 would be parallel to 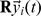 and so the scalar product in equation (9) will be 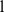 for an expansion and 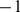 for a contraction.

### Normalization of the correlation function

In the study of flocks [19], we normalized 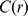 by its limiting value for 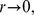 which is equivalent dividing it by the value in the first bin. In that way the normalized correlation function tends to 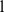 for 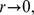 so that its value is amplified. In the study of flocks we were only looking at the correlation length, which is not altered by such a normalization. However, here we will be interested in both the range and the intensity of the correlation, so we must not amplify artificially the correlation signal. Normalising the fluctuations as in (1) is equivalent normalising the correlation function by its value at *exactly* 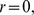 i.e. for 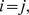 which is different from its limit for 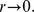


### Non-interacting Harmonic Swarm

The NHS is an elementary model of non-interacting particles performing a random walk in a three-dimensional harmonic potential. The dynamics of each particle is defined by the Langevin equation,
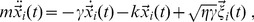
(10)where 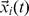 is the position of the 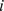-th particle at time 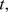 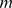 is the mass, 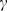 the friction coefficient, 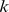 the harmonic constant and 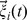 is a random vector with zero mean and unit variance, 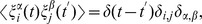 with 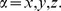 Clearly, in this model there is no interaction between particles. The parameter 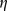 tunes the strength of the noise. The equation of motion is integrated with the Euler method [52]. We simulated the NHS in the critically damped regime (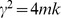), which gives the best similarity to natural swarms. The number of particles 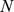 is set equal to that of the natural swarm we want to compare it with. Parameters have been tuned to have a ratio between the distance travelled by a particle in one time step (frame) and the nearest neighbour distance comparable to natural swarms, 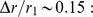 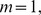 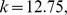 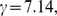 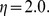


### Definition of P-value

Let us define a data set as a collection of 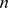 pairs of variables, 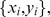 with 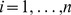 (for example, the susceptibility as a function of the rescaled nearest neighbour distance - Fig. 4). The null hypothesis is that 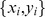 are independent variables. Let us call 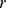 the Spearman's rank correlation coefficient for a set of 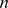 data and 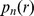 the probability distribution of 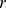 in the case of 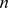 pairs of *independent* variables. Given the empirical data, we calculate the Spearman's rank correlation coefficient and get a certain value, 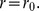 The P-value is defined as the probability that the statistical test we are using (Spearman) gives a result at least as extreme as the one actually observed, *provided that the null hypothesis is true*. Hence, the P-value is given by,
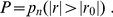
(11)


Basically, the P-value is telling us how likely it is that the degree of correlation that we observe is just the result of chance. In absence of an *a priori* model of the noise, we estimate 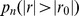 by a permutation test [53], [54]: using the original paired data, 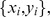 we randomly redefine the pairs to create a new data set 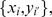 where the 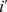 are a permutation of the set 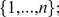 we calculate the Spearman's rank correlation coefficient 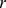 of this new randomized data set; we iterate this permutation 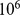 times; we compute the fraction of permutations that give 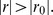 This fraction is equal to the P-value of the data set under consideration [53].

### Vicsek model

We performed numerical simulations of the Vicsek model in 3d [35]–[38], [55]. The direction of particle 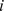 at time 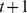 is the average direction of all particles within a sphere of radius 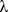 around 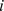 (including 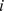 itself). The parameter 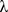 is the metric radius of interaction. The resulting direction of motion is then perturbed with a random rotation (noise). Natural swarms are known to form close to a marker and to keep a stationary position with respect to it [13]. To mimic this behaviour we modified the Vicsek model by adding an external harmonic force equal for all particles. This potential also grants cohesion, without the need to introduce an inter-individual attraction force [4], [16], [27].

The update equation for velocities is therefore given by,
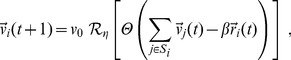
(12)where 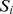 is the spherical neighbourhood of radius 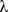 centred around 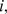 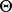 is the normalization operator, 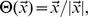 and 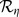 performs a random rotation uniformly distributed around the argument vector with maximum amplitude of 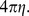 The term 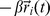 is the harmonic force directed towards the origin. For 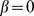 we recover the standard Vicsek model. The update equation for the positions is, 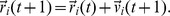 Thanks to the central force we can use open boundary conditions. All particles have fixed velocity modulus 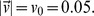 Each simulation has a duration of 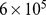 time steps, with initial conditions consisting in uniformly distributed positions in a sphere and uniformly distributed directions in the 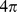 solid angle. After a transient of 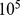 time steps, we saved 500 configurations at intervals of 1000 time steps in order to have configurations with velocity fluctuations uncorrelated in time. The control parameter of interest is 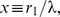 where 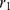 is the nearest neighbour distance, which is tuned by 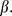 The model displays a transition to an ordered phase when 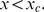 We studied the susceptibility 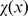 for different values of 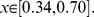 To observe the power-law behaviour of 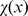 predicted by the model we performed standard finite-size scaling [38]: at each fixed value of the system' size 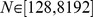 we calculated 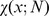 and worked out the maximum of the susceptibility 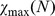 and its position 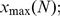 we finally plotted 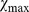 vs. 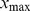 parametrically in 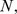 to obtain the function 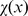 in Fig. 5. The noise, 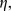 affects the position of the transition point 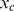 [35]–[37], but this is irrelevant for us, because we do not use any quantitative result from the model to infer the biological parameters of real swarms. The data reported in Fig. 5 have 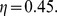


### Percolation threshold

For each frame we run a clustering algorithm with scale 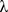 [56]: two points are connected when their distance is lower than 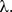 For each value of 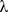 we compute the ratio 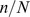 between the number of objects in the largest cluster and the total number of objects in the swarm (Fig. 6). The percolation threshold, 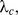 is defined as the point where a giant cluster, i.e. a cluster with size of the same order as the entire system, forms [42]. We define 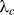 as the point where 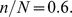 The percolation threshold scales with the nearest neighbour distance, 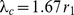 (Fig. 6). Strictly speaking, the percolation argument only holds at equilibrium, because in a system where particles are self-propelled there may be order even at low density [36]. However, at low values of the noise, we still expect the percolation argument to give a reasonable, albeit crude, estimate of the perception range.

## Supporting Information

Datafile S1 Datafile S1 refers to a swarm of *Chironomidae, Cladotanytarsus atridorsum*. Data from this file were used to produce the panels in the first two rows of the left column of Fig. 3.
(DAT)

Datafile S2 Datafile S2 refers to a swarm of *Chironomidae, Corynoneura scutellata*. Data from this file were used to produce the panels in the first two rows of the central column of Fig. 3.
(DAT)

Datafile S3 Datafile S3 refers to a swarm of *Ceratopogonidae, Dasyhelea flavifrons*. Data from this file were used to produce the panels in the first two rows of the right column of Fig. 3.
(DAT)

Text S1 In the text file, helpful details about the importance and the interpretation of the connected correlation function and of the susceptibility are reported together with a table summarizing the main properties of the analysed swarming events. The three supporting data files report the positions and the velocities used to compute the correlation functions for a single instant of time. The three files refer to the three different analysed species of midges. In each file, for each pair of midges in the swarm, the mutual distance and the scalar product between their dimensionless velocity fluctuations (see Eq.1) are reported: the distances in meters in the first column, the dimensionless scalar products in the second column.
(PDF)

Video S1Wild swarm of roughly 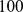 midges in the field (Diptera:Ceratopogonidae). The swarm has been video recorded at 170 frames per second, with a resolution of 4Mpix, using an IDT-M5 camera.(AVI)

Video S2 Three dimensional visualization of the same natural swarm as in Video S1. This 3d reconstruction has been obtained by means of our dynamical tracking algorithm based on the trifocal experimental technique.
(AVI)

Video S3 Three dimensional visualization of a numerically simulated swarm of non-interacting particles in a harmonic potential (NHS, Non-interacting Harmonic Swarm). The number of ‘midges’ in the NHS is the same as in Video S2.
(AVI)

Video S4Three dimensional visualization of a numerically simulated swarm obtained using Vicsek model with the addition of an harmonic attraction force towards the origin. The video refers to a swarm in the ordered phase, with the polarization equal to 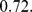 The number of “midges” in the swarm is 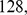 the harmonic constant 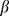 is equal to 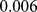 while the simulation noise 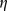 is equal to 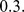

(MP4)

Video S5Three dimensional visualization of a numerically simulated swarm obtained using Vicsek model with the addition of an harmonic attraction force towards the origin. The video refers to a swarm in the disordered phase, with the polarization equal to 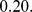 The number of “midges” in the swarm is 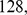 the harmonic constant 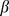 is equal to 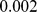 while the simulation noise 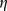 is equal to 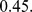

(MP4)
