# Supplementary material for: Collective Behaviour without Collective Order in Wild Swarms of Midges
Source: PLoS Comput Biol. 2014 Jul 24;10(7):e1003697. doi: 10.1371/journal.pcbi.1003697 (PMC4109845; doi:10.1371/journal.pcbi.1003697)
Supplement: Text S1 — In the text file, helpful details about the importance and the interpretation of the connected correlation function and of the susceptibility are reported together with a table summarizing the main properties of the analysed swarming events. The three supporting data files report the positions and the velocities used to compute the correlation functions for a single instant of time. The three files refer to the three different analysed species of midges. In each file, for each pair of midges in the swarm, the mutual distance and the scalar product between their dimensionless velocity fluctuations (see Eq.1) are reported: the distances in meters in the first column, the dimensionless scalar products in the second column. (PDF) [file pcbi.1003697.s004.pdf]

SUPPORTING INFORMATION

1 Connected vs non-connected correlation

The most basic kind of correlation one can measure in a system is the scalar product of the velocities of two different individuals, $\vec{v}_i \cdot \vec{v}_j$. This quantity is large if velocities are pointing in the same direction and low if they are uncorrelated. This is what is called *non-connected* correlation, and it has a problem: its value is trivially dominated by the *mean* motion of the system. Imagine that a gust of wind shifts the entire swarm, so that each midges's velocity is dominated by the wind speed. As an effect of the wind, midges i and j would be moving nearly parallel to each other, so that the non-connected correlation would be high. This, however, is simply an effect of the wind, and it is not a genuine sign of correlation, nor of interaction between the individuals. The same thing would happen in a system of uncorrelated particles put in rotational motion around an axis: velocities of nearby particle are mostly parallel as a mere effect of the overall rotation.

These examples show that, in order to get information about the *bona fide* interaction between individuals, we need to compute the correlation between the *fluctuations* around the mean motion of the system. In other words, what we want to detect is to what extent the individual changes of behaviour with respect to the global behaviour of the system are correlated. This is what the *connected* correlation does and it is the only reliable measure of correlation in a system. The presence of a non-connected correlation is not in general proof of anything at the level of the interaction, as the wind example clearly shows. On the other hand, the presence of non-zero connected correlation in a system is an unambiguous proof that there is interaction, and strong enough to produce collective effects.

Let us explain this fact by using again the wind example: if the only reason for the overall motion of the swarm is the wind, then the connected correlation function vanishes, showing that there is no real self-organization in the system; if, on the other hand, the overall group motion is due to self-organization, as it happens in starling flocks, then the connected correlation function *does not* vanish [1], because not only the directions of motions are correlated, but also the fluctuations with respect to these directions are correlated. Hence, the connected correlation function is able to distinguish these two drastically different cases (collective motion due to an external cause vs. self-organized collective motion), whereas the non-connected correlation would be large in both cases. This is the reason why the non-connected correlation function is the wrong tool to probe self-organization.

To compute the connected correlation function we therefore need to identify the collective modes of motion of the system and subtract them from the individual motion (see Methods). In this way we obtain the velocity fluctuation, namely the velocity of midge i in a reference frame that not only is co-moving with the centre of mass, but also rotating and expanding/contracting as the whole swarm. Therefore, what is left is the deviation of i from the mean group motion, which is the only quantity that is safe to correlate.

It is very important to realize that an error or an artefact in computing the fluctuations can lead to spurious values of the correlation. As an example, consider two different and unrelated swarms moving in opposite directions, because of some weird fluctuation of the wind. If we fail to notice that these are *two* systems and analyse our data as if they were *one*, we get a zero net motion of the centre of mass. Hence, the velocity fluctuations are equal to the full velocities, and we are effectively computing a non-connected correlation, rather than a connected one, giving the delusion of very large correlation.

In the main text we show that swarms are mostly disordered. However, the fact that order parameters are low on average, does not mean that we can use the full velocities to compute the correlation function. As we have already said, a brief gust of wind can push the non-connected correlation function to very high values. In this study, we are not investigating the origin of the order parameters fluctuations, but we focus on correlations. Hence, we have to be sure that correlation is computed in a way to avoid any spurious signal from the collective modes.

2 Susceptibility, response and correlation

In a stationary system, it can be proven [2] that the susceptibility is equal to the collective response of the system to uniform external perturbations. Maximum entropy calculations [3] show that the stationary probability distribution of the velocities in systems where there is an alignment interaction is given by,

$$P(v) = \frac{1}{Z}\, e^{J\sum_{i,j}\vec{v}_i\cdot\vec{v}_j} \ , \tag{1}$$

where J is the strength of the interaction (depending on the distance r in a metric system) and Z is a normalizing factor (the partition function),

$$Z = \int Dv\ e^{J\sum_{i,j}\vec{v}_i\cdot\vec{v}_j} \ . \tag{2}$$

where Dv indicates that Z is a volume integral over the space of the velocities, $Dv = \prod_i \delta v_i$. If an external perturbation (or field) h couples uniformly to all velocities, this distribution gets modified as,

$$P(v) = \frac{1}{Z(h)}\, e^{J\sum_{i,j}\vec{v}_i\cdot\vec{v}_j + \vec{h}\cdot\sum_i\vec{v}_i} \ . \tag{3}$$

Now we ask what is the collective response χ of the system to a small variation of the perturbation h. To answer this question we calculate the variation of the global order parameter, i.e. of the space average of the velocity, under a small variation of h. We have,

$$\begin{aligned}
\chi &= \frac{\partial}{\partial h}\langle\frac{1}{N}\sum_k v_k\rangle \\
&= \frac{\partial}{\partial h}\int Dv\ P(v)\frac{1}{N}\sum_k v_k \\
&= \frac{1}{N}\sum_{i,k}\int DvP(v)v_k v_i - \int DvP(v)v_i\int DvP(v)v_k \\
&= \frac{1}{N}\sum_{i,k}\langle v_k v_i\rangle - \langle v_i\rangle\langle v_k\rangle = \frac{1}{N}\sum_{i,k}\langle\delta v_k\ \delta v_i\rangle \ ,
\end{aligned} \tag{4}$$

where we have disregarded the vectorial nature of the quantities not to burden the notation, and where we have defined,

$$\langle f(v)\rangle = \int DvP(v)f(v) \ . \tag{5}$$

Apart from the missing normalization, needed to make χ dimensionless, the quantity in (4) is exactly the susceptibility defined in the main text, equation (2).

Let us now analyse in detail the relation between correlation function and susceptibility in a finite size system, where instead of the ensemble averages, $\langle\cdot\rangle$, we can only perform space averages. From equations (1) and (2) in main text, we obtain:

$$Q(r) = \frac{1}{N}\int_0^r dr'\sum_{i\neq j}^N\ \delta(r'-r_{ij})\,C(r') \ . \tag{6}$$

If we make the hypothesis that mass fluctuations are not strong, we can write,

$$\frac{1}{N}\sum_{i\neq j}^N\ \delta(r'-r_{ij}) \sim 4\pi x^2\rho \ , \tag{7}$$

where ρ is the density. Hence, we get,

$$Q(r) = \frac{3}{r_1^3}\int_0^r dr'\ r'^2\,C(r') \ , \tag{8}$$

where we have used the simple relationship between density and nearest neighbours distance, $4\pi\rho = 3/r_1^3$. In an infinitely large system, the bulk susceptibility is simply, $\chi_\infty = Q(r\to\infty)$, that is the full volume integral of the connected correlation function. In a finite size system, however, due to the constraint, $\sum_i \delta\vec{\varphi}_i = 0$, we must have,

$$Q(r=L) = -1 \ , \tag{9}$$

for all systems, be they natural or synthetic, irrespective of the amount of real correlation. This relation is simply the mathematical consequence of the way velocity fluctuations are defined. Therefore, in a finite system the susceptibility can be estimated as the maximum value reached by $Q(r)$ (this maximum is a lower bound for the bulk susceptibility). We know that, $C(r_0) = 0$, so that the function $Q(r)$ reaches its maximum at $r = r_0$. Hence the finite size susceptibility is given by,

$$\chi = Q(r_0) = \frac{3}{r_1^3} \int_0^{r_0} dr\, r^2\, C(r) \ . \tag{10}$$

Species	Event label	N	Duration (s)	l (mm)	r_1 (m)	r_0 (m)	$\lvert\vec{v}\rvert$ (m/s)	χ	ϕ
Corynoneura scutellata - CS (Diptera:Chironomidae)	20110906_A3	138	2.0	1.5	0.029	0.094	0.12	0.78	0.17
	20110908_A1	119	4.4	1.1	0.036	0.105	0.13	0.46	0.27
	20110909_A3	312	2.7	1.5	0.026	0.138	0.12	2.58	0.22
Cladotanytarsus atridorsum - CA (Diptera:Chironomidae)	20110930_A1	173	5.9	2.4	0.057	0.228	0.23	1.48	0.31
	20110930_A2	99	5.9	2.4	0.063	0.223	0.15	1.08	0.20
	20111011_A1	131	5.9	2.4	0.075	0.272	0.11	0.65	0.17
	20120828_A1	89	6.3	2.5	0.062	0.188	0.17	0.48	0.22
	20120907_A1	169	3.2	1.9	0.062	0.330	0.13	1.72	0.20
	20120910_A1	219	1.7	2.4	0.047	0.221	0.19	2.25	0.27
	20120917_A1	192	0.36	2.2	0.043	0.219	0.12	2.09	0.14
	20120917_A3	607	4.23	2.2	0.033	0.259	0.10	5.57	0.15
	20120918_A2	69	15.8	1.7	0.060	0.174	0.15	0.64	0.23
	20120918_A3	214	0.89	1.7	0.041	0.230	0.20	2.04	0.36
Dasyhelea flavifrons - DF (Diptera:Ceratopogonidae)	20110511_A2	279	0.9	2.3	0.053	0.248	0.20	1.25	0.35
	20120702_A1	98	2.1	2.0	0.062	0.162	0.14	0.69	0.20
	20120702_A2	111	7.3	2.0	0.056	0.169	0.13	0.88	0.18
	20120702_A3	80	10.0	2.0	0.060	0.170	0.12	0.32	0.20
	20120703_A2	167	4.4	1.8	0.046	0.140	0.07	0.52	0.12
	20120704_A1	152	10.0	1.7	0.050	0.154	0.09	0.63	0.15
	20120704_A2	154	5.3	1.7	0.053	0.160	0.08	0.61	0.13
	20120705_A1	188	5.9	1.8	0.055	0.182	0.12	0.92	0.20

Table 1. Swarm data. Each line represents a different swarming event (acquisition). N is the number of individuals in the swarm, r_1 the time average of the nearest neighbour distance in the particular acquisition, r_0 the average correlation length, χ the average susceptibility and ϕ the average polarization.

References

1. Cavagna A, Cimarelli A, Giardina I, Parisi G, Santagati R, Stefanini F, Viale M (2010) Scale-free correlations in starling flocks. Proc Natl Acad Sci U S A 107: 11865-11870.

2. Binney JJ, Dowrick NJ, Fisher AJ, Newman M (1992) The theory of critical phenomena: an introduction to the renormalization group. Oxford University Press, Oxford.

3. Bialek W, Cavagna A, Giardina I, Mora T, Silvestri E, Viale M, Walczak AM (2012) Statistical mechanics for natural flocks of birds. Proc Natl Acad Sci U S A 109: 4786-4791.

4. Cardy J (1996) Scaling and renormalization in statistical physics. Cambridge Lecture Notes in Physics, Cambridge University Press, Cambridge.

5. Toner J, Tu Y (1998) Flocks, herds, and schools: A quantitative theory of flocking. Phys Rev E 58: 4828-4858.

6. Cavagna A, Giardina I, Ginelli F (2013) Boundary information inflow enhances correlation in flocking. Phys Rev Lett 110: 168107.
